# Object Relocation Visual Tracking Based on Histogram Filter and Siamese Network in Intelligent Transportation

**DOI:** 10.3390/s22228591

**Published:** 2022-11-08

**Authors:** Jianlong Zhang, Yifan Liu, Qiao Li, Ci He, Bin Wang, Tianhong Wang

**Affiliations:** 1 School of Electronic Engineering, Xidian University, Xi’an 710071, China; 2 Science and Technology on Communication Networks Laboratory, Shijiazhuang 050000, China

**Keywords:** single object tracking, Siamese network, dynamic template set, match filter

## Abstract

Target detection and tracking algorithms are one of the key technologies in the field of autonomous driving in intelligent transportation, providing important sensing capabilities for vehicle localization and path planning. Siamese network-based trackers formulate the visual tracking mission as an image-matching process by regression and classification branches, which simplifies the network structure and improves the tracking accuracy. However, there remain many problems, as described below. (1) The lightweight neural networks decrease the feature representation ability. It is easy for the tracker to fail under the disturbing distractors (e.g., deformation and similar objects) or large changes in the viewing angle. (2) The tracker cannot adapt to variations of the object. (3) The tracker cannot reposition the object that has failed to track. To address these issues, we first propose a novel match filter arbiter based on the Euclidean distance histogram between the centers of multiple candidate objects to automatically determine whether the tracker fails. Secondly, the Hopcroft–Karp algorithm is introduced to select the winners from the dynamic template set through the backtracking process, and object relocation is achieved by comparing the Gradient Magnitude Similarity Deviation between the template and the winners. The experiments show that our method obtains better performance on several tracking benchmarks, i.e., OTB100, VOT2018, GOT-10k, and LaSOT, compared with state-of-the-art methods.

## 1. Introduction

In recent years, autonomous driving has been considered one of the most promising areas of automotive research for research and development. Vision tracking technology has a significant role to play in the field of autonomous driving. Vision sensors are used to dynamically track the target vehicle during the vehicle’s driving process to achieve information interaction between vehicles such as location and driving status, as well as the vehicle’s road condition perception of the environment.

Although vision tracking technology has made significant progress in recent years, factors such as interference targets, appearance deformation, and motion blur can seriously affect the performance and robustness of tracking algorithms in practical application scenarios.

Traditional trackers, such as the correlation filter tracker represented by the correlation filter KCF [1] and CSK [2] tracker with a circular kernel matrix structure, have excellent tracking speed and allow fast online updating of the filter weights. However, the robustness of these trackers is not satisfactory due to the weak semantic information of the artificial features. With the development of deep neural networks, C-COT [3] and MDnet [4] have improved the accuracy of trackers by replacing the traditional manual features with deep features.

Early visual tracking methods can be divided into two categories according to the tracking mode, namely, the generative model and the discriminative model. With the development of deep learning, visual tracking methods based on deep learning have gradually become mainstream.

Visual tracking is focused on the research of the generative model, such as the optical flow method [5], particle filter [6], and mean-shift algorithm [7]. An object model or extracted object features are first established, and then similar features are searched for in subsequent frames. However, the background information of the image is not fully considered. Hence, it is very limited in terms of describing the object through a single mathematical model.

Considering the object and background information at the same time, the discriminative model regards the tracking process as a classification or regression problem, and the purpose is to find a discriminant function to separate the object from the background, so as to realize the tracking of the object. The evaluation of the algorithm [8] found that the performance of the tracker could be greatly improved by introducing background information into the tracking model. Therefore, various classifiers were introduced into the visual tracking field. Avidan [9] used support vector machines [10] to distinguish the background and the object, but it is easy to lose the object due to the selected feature being based on a single pixel. TLD [11] used online Ferns [12] to detect objects, while using an online random forest algorithm [13] to track objects. In 2010, cross-correlation was introduced into visual tracking [14]. As a discriminative method, it showed better performance in terms of speed and accuracy. STRCF [15] considered both spatial regularization and time regularization. It could successfully track objects under occlusion and could adapt to larger appearance changes.

The introduction of deep features enhances the feature representation capability of the tracker. HCF [16] utilized the deep and shallow features of the VGG [17] network and incorporated the relevant filters to obtain good tracking performance.

Recently, Siamese networks based on trackers have received significant attention for their balance between high speed and accuracy [18,19]. SINT++ [20] used the positive sample generation network to obtain diverse sample images, by which the robustness of the tracker is improved. SA-Siam [21] utilized two networks to obtain semantic features and appearance features, respectively, and introduced the attention mechanism and feature fusion into the semantic branch network. SiamMask [22] solved the problem of visual tracking and object segmentation at the same time, and introduced the segmentation branch to obtain an accurate mask. 

In addition, the development of adversarial training [23] has improved the accuracy of Siamese network-based trackers and has been applied to intelligent transportation [24], autonomous driving [25], and other domains [26,27]. These trackers first perform feature extraction using a Siamese network, and then exploit a tracking-head network to localize objects from the similarity map. The head network between the search branch and the template branch increases the speed and reduces the overfitting owing to frequent updates of the template. The architecture of these trackers consists of three parts, namely, a Siamese backbone network for template-region and search-region feature extraction, a similarity-matching component for search and template branch information embedding, and a tracking head for information decoding from similarity maps. SiamFC [19] obtains features through a Siamese backbone and introduces a correlation layer to compute the similarity scores of feature maps to localize an object with a lightweight architecture that does not need any updating of the model parameters. SiamFC works efficiently at 86 FPS with high accuracy. RASNet [28] combined a Siamese network with several attention mechanisms to emphasize more relevant parameters to the object. However, these trackers require a multiscale transformation to deal with scale variations. In order to obtain a more accurate and robust result, SiamRPN [29] introduced the RPN [30] into the SiamFC and achieved high accuracy. Both SiamRPN++ [31] and SiamDW [32] reduced the effect of adverse factors (e.g., padding) and decreased the impact of border effects in distinct ways. They introduced deeper neural networks, e.g., ResNet [33], into visual tracking. The anchor-based tracker requires tedious and heuristic configurations, but prior parameters are difficult to fit all objects, which reduce the tracker accuracy. Some anchorless trackers, such as SiamFC++ [34] and SiamCAR [35], took one or more heads to directly predict the position of the object and regress the bounding boxes from the similarity map. Siamese network-based trackers have made great developments; however, the following drawbacks still exist. (1) Owing to the constraints of strict translation invariance and real-time requirements, lightweight neural networks lead to inadequate feature representation. When distractors are presented in the vicinity of the object, it is difficult for the tracker to distinguish which is the right object. (2) Due to the lack of an efficient template update strategy, the single template cannot suit changes in object features, which causes tracking failure when there are large appearance distortions or perspective changes. (3) Due to the lack of an effective arbiter-corrector module, the tracker cannot detect tracking failures, and cannot relocate the object once the object is lost and restart tracking.

To overcome the above problems, we proposed a SiamFC++-based object relocation tracker. The main contributions of the work are as follows.

- We designed a matching filter arbiter with a hierarchical architecture based on the distance histogram of the candidate objects, which can accurately and quickly find the failure.
- We propose an efficient corrector that generates a template set by backtracking. The corrector relocates the object by Gradient Magnitude Similarity Deviation (GMSD) and the assignment algorithm measurement to increase the tracker’s resistibility to interference.
- Experiments on several challenging benchmarks including VOT-18, GOT-10k, OTB-100, and LaSOT have shown that our proposed tracker is superior to many state-of-the-art trackers.

The remainder of this paper is organized as follows. The section Materials and Methods introduces the related work of visual tracking and describes the principle and implementation of our tracker. The Results section evaluates and analyzes the experimental results. Finally, we summarize our work in the Conclusion section.

## 2. Materials and Methods

**Figure 1:**
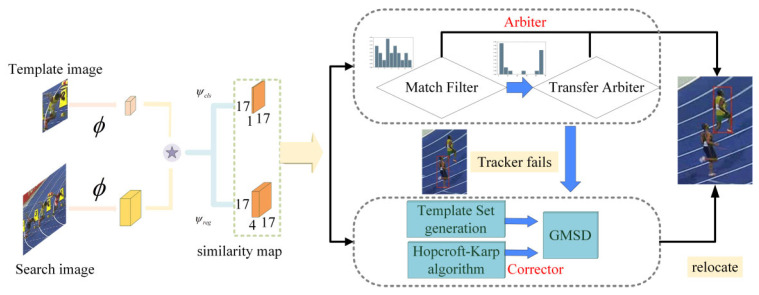
Framework of the proposed tracker architecture.

As shown in Figure 1, the framework of our tracker consists of SiamFC++, the arbiter, and the corrector. Firstly, SiamFC++ produces a similarity map according to the search branch and the template branch. Secondly, the arbiter consists of the matching filter arbiter and the transfer arbiter, designed to determine whether the tracking fails. Finally, the corrector implements the repositioning of the tracker, which includes template set update, assignment algorithm, and GMSD score.

### 2.1. SiamFC++

**Figure 2:**
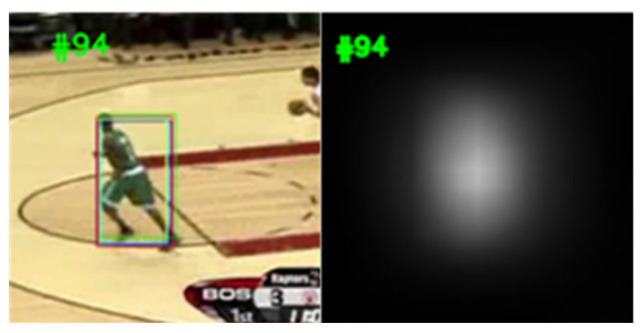
Similarity map without distractor.

**Figure 3:**
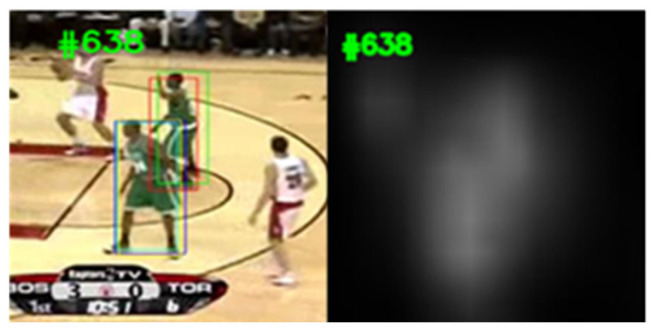
Similarity map with distractor.

SiamFC++ introduces four principles for designing trackers and the anchor-free structure to reduce the prior knowledge, and combines classification and regression branches to increase the tracking accuracy. SiamFC++ extracts the deep feature map of the search regions and the template regions, respectively, through backbone network, and inputs the feature maps into the regression head and classification head, respectively, to obtain the similarity map, where the highest scoring position represents the object position. The similarity map is the degree of similarity between different positions of the search image and the template. As shown in Figure 2 and Figure 3, the bounding boxes are the location of the object with different similarity scores. The similarity map has only one center, so there is no distractor round the object (Figure 2). When the similarity score of the distractor and the object is similar, the similarity map has two centers (Figure 3). Because SiamFC++ focuses on both the object and the distractor, the results may shift from objects to distractors. Occlusion, deformation, scale variation, and distractor are the main disruptive factors in tracking datasets. Table 1 shows the percent of SiamFC++ fails factors on the VOT-18 dataset. It is clear that 60% of the failures are caused by distractor and deformation; therefore, the performance of the tracker could be promoted if we could determine the failures and relocate the object. For this purpose, we designed a system that contains two modules, namely, the arbiter and the corrector. In order to explain its mathematical principle, we let N be the length of the video frames and $S_{j}^{i}$ the similarity score of the bounding box with the similarity rank j in the i-th frame. $R=\{R_{1},R_{2},\;\cdot \;\cdot \;\cdot \;,\;R_{i},\;\cdot \;\cdot \;\cdot \;|\;1\;\leq \;i\;\leq \;N\}\;$ is the bounding box set of the object. $D=\{D_{1}^{f},D_{2}^{f},\;\cdot \;\cdot \;\cdot \;,\;D_{n}^{f},\;\cdot \;\cdot \;\cdot \;|S_{1}^{f}>S_{2}^{f}>\ldots >S_{n}^{f}\}$ is the candidate object set in the f-th frame. $T=\left\{T_{1},T_{2},\;\cdot \;\cdot \;\cdot \;,\;T_{p-1},T_{p}\;\right\}\;$ is the templates set, where $T_{p}$ is the p-th template. $J\in \left\{J_{1},J_{2},\;\cdot \;\cdot \;\cdot \;,J_{m}\;\right\}\;$ is the set of tracking failure frames, which indicates that the tracker fails in the $J_{i}$ frame. For the convenience of reading, we list the symbols used in this paper in Table 2.

### 2.2. Arbiter

Herein, the purpose of the arbiter is to determine whether the tracker fails. We need to reposition the object once the tracking fails. The main matching filter arbiter based on the candidate object histogram determines whether distractors exist. The transfer arbiter determines whether the object is transferred based on the change of the object in relative position, and finally, both together determine whether the tracker is not working.

#### 2.2.1. Match Filter

**Figure 4:**
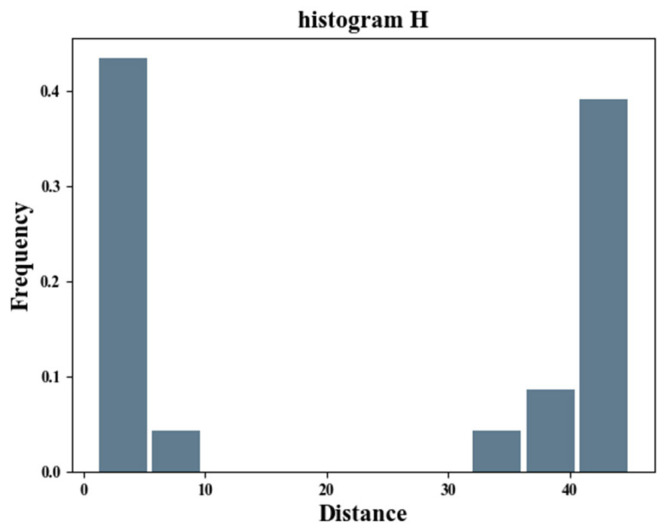
Histogram H.

Considering the observation that the similarity map shows two highlighted areas corresponding to the distractor and the object, respectively, when the distractor is presented, we attempt to arbitrate the existence of the distractor using the histogram of the distance between the region centroids. Let $L_{ij}$ be the Euclidean distance between the object centers where $\left(x_{i}^{’},y_{i}^{’}\right)$ is the center of $D_{i}$. The specific expression of $L_{ij}$ is shown in Equation (1). The distance is small among similar candidate objects, while it is large between different types. Therefore, the distance shows a trend of bipolar distribution. The histogram H is similar to a band stop filter, as shown in Figure 4. Equation (2) is a histogram, i.e.,:

(1) $$L_{ij}=\sqrt{{\left(y_{i}^{’}-y_{j}^{’}\right)}^{2}+{\left(x_{i}^{’}-x_{j}^{’}\right)}^{2}}$$

(2) $$H=\left\{H_{1},H_{2},\;\cdot \;\cdot \;\cdot \;,H_{k}\;\right\},$$

where $H_{i}$ is the frequency of the i-th bin, k is the number of histogram bins (k ≥ 10), and the total frequency is $\;C_{2}^{k}$. We input H into the band stop filter F to obtain the filter output Z. Equation (3) is a discrete band stop filter. The calculation of Z is shown in Equation (4). When the output is greater than the threshold T, it indicates that there are interferences in the image.

(3) $$F=\left\{F_{1},F_{2},\;\cdot \;\cdot \;\cdot \;,F_{k}\;\right\}$$

(4) $$\begin{array}{c} Z=\overset{k}{\underset{i=1}{\sum }}H_{i}\times F_{i} \end{array}$$

#### 2.2.2. Transfer Arbiter

**Figure 5:**
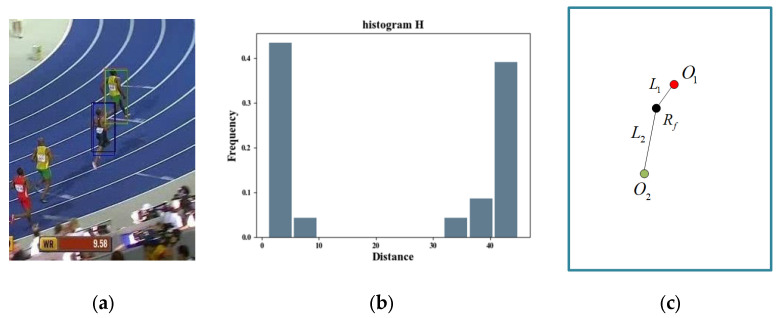
Transfer arbiter. (**a**) shows the distribution of candidate targets for the current scene; (**b**) is the result of histogram statistics of the distance between candidate frames; (**c**) shows the tracking results and the center distance of similar objects schematically.

The matching filter arbiter could determine whether the distractor exists, but it could not determine whether the object is displaced by the distractor. As shown in Figure 5a, the red border is the object position, but the distance histogram also takes the shape of a band stop filter, as shown in Figure 5b. Therefore, it is difficult to determine whether the tracker fails if only using the matching filter arbiter. It is known that the change in the distance between the objects in adjoining frames is less than the distance between the distractor and the object in the general tracking scene. The relative distance changes when the tracking result is moved to the distractor. It is possible to further determine whether the tracker fails based on the changes in the relative locations of the distractor and the object in the adjacent frames.

**Figure 6:**
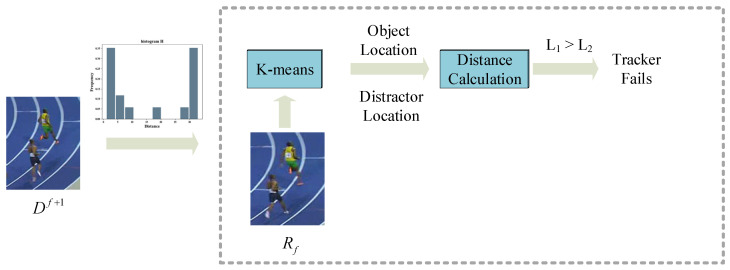
Transfer arbiter framework.

The framework of the transfer arbiter is shown in Figure 6. When the f+1 frame passes through the matching filter arbiter, it is classified via the K-means algorithm, where K=2. As shown in Figure 5c, the center closer to $\left(x_{1},y_{1}\right)$ is the object position $O_{1}$, and the other center is the distractor position $O_{2}$. $L_{1}$ is the Euclidean distance between $R_{f}$ and $O_{1}$. $L_{2}$ represents the distance between $R_{f}$ and $O_{2}$. The expressions of$\;L_{1}$ and $L_{2}$ are shown in Equation (5) and Equation (6), respectively. The procedure of transfer arbiter is as follows. In order to demonstrate the effectiveness of the proposed arbiter, we calculate the tracking invalid accuracy ratio (TIAR) on every dataset, which is the percentage of correct judgments among tracking failure sequences. Although the length of the GOT-10k video sequences is relatively long, it does not perform satisfactorily and the arbiter still works in 40% of the failure scenarios.

(5) $$L_{1}=\sqrt{{\left(x_{R_{f}}-x_{O_{1}}\right)}^{2}+{\left(y_{R_{f}}-y_{O_{1}}\right)}^{2}}$$

(6) $$L_{2}=\sqrt{{\left(x_{R_{f}}-x_{O_{2}}\right)}^{2}+{\left(y_{R_{f}}-y_{O_{2}}\right)}^{2}}$$

### 2.3. Corrector

The object needs to be relocated once the tracker fails. We propose a corrector consisting of dynamic template update, assignment algorithm, and GMSD. Updated by the similarity backtracking, the template set is to find the previous tracking results. The assignment algorithm is used for selecting the winner set, which is similar to the object. Finally, the object is relocated by computing the GMSD between the template set and winner set.

#### 2.3.1. Dynamic Template Set

In long-term tracking, a single template cannot handle the changes of object appearance, such as: (1) when the object appearance changes gradually, the error accumulates and finally the object cannot match the template well, and (2) when the object appearance changes suddenly and drastically, the object is very different from the template. Therefore, we propose a template update procedure that automatically adds the result different from the template into the template set, so that the diversity of the template set can be enriched. The template update mechanism is to find the target with the lowest similarity score when the target is lost by retracing the tracking failure process, i.e., the image with the largest change in appearance during the tracking process, and add this target to the template set.

**Figure 7:**
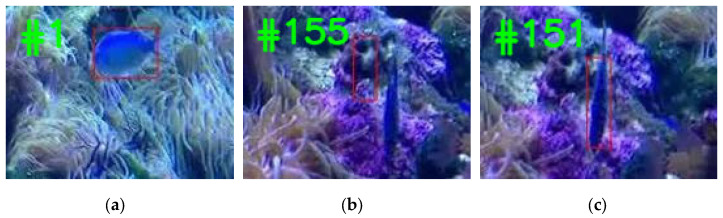
Dynamic template set update. (**a**) shows the original template of the tracking process; (**b**) shows the scene where the appearance of the target has changed; (**c**) shows the updated template.

The process of generating the template set is shown in Figure 7. The template image is shown in (a). When the appearance of the object in (b) changes significantly, the tracker is disabled. Meanwhile, the object in frame 151 in (c) is added to the updated template set. The template will be more similar to the object in the next frames.

#### 2.3.2. Assignment Algorithm

**Figure 8:**
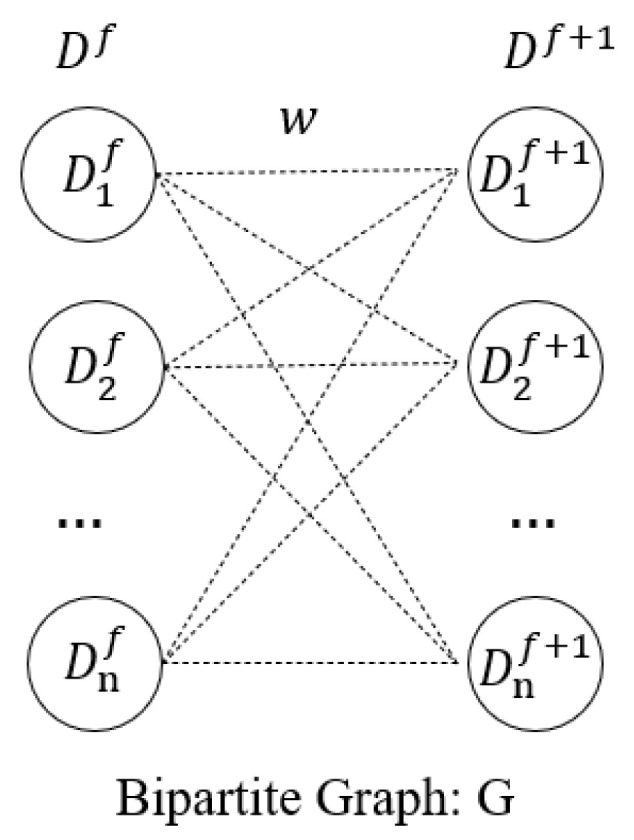
Bipartite graph-matching model.

Since there are lots of candidate objects, the corrector utilizes the Hopcroft–Karp algorithm [36] to select the set of winners with high similarity from the candidate objects, and calculates the GMSD between the winners and the template to reduce the computation and improve the speed. The Hopcroft–Karp algorithm is used to realize bipartite graph matching. Compared with the Kuhn–Munkres [37] algorithm, it looks for multiple augmentation paths at once. This can further decrease the time complexity and achieve the optimal complete match. The bipartite graph-matching model is shown in Figure 8. The matching process is as follows.

(1) Take an initial match *M* from G=(X, Y ;ω). The weight ω calculation between different vertex is shown in Equation (7);
(2) While there exists an augmenting path P, remove the matching edges of *P* from *M* and add non-matching edges of P to M (this increases the size of M by 1 as P starts and ends with a free vertex, i.e., a node that is not part of the matching);
(3) Return M.
  (7) $$\begin{array}{c} \omega \left(D_{i}^{f},D_{i}^{f+1}\right)=\phi \left(D_{i}^{f}\right)⊗\phi \left(D_{i}^{f+1}\right) \end{array}$$
  where ϕ(·) is the Siamese backbone for feature extraction and ⊗ is the cross-correlation operator.

We obtained a complete match of $D_{f}$,$\;D_{f+1}$ using the Hopcroft–Karp algorithm. Define $C=\left\{C_{1}^{f+1},\;\cdot \;\cdot \;\cdot \;,C_{q}^{f+1}\;\right\}$ as the winner set, $C_{i}^{f+1}$ is the candidate object that $D_{i}^{f}$ matches, and q is the number of winners.

#### 2.3.3. GMSD Relocation

Since the backbone of SiamFC++ has difficulty in distinguishing the distractor from the object, it is essential to choose another efficient algorithm to restart the tracker. We introduce the gradient magnitude similarity deviation (GMSD) to relocate the object. GMSD can distinguish the object by its appearance and structure, and only uses the gradient magnitude as a feature to generate a highly accurate score. It can precisely locate the objects that are similar to the template in the case of similar semantic information. The calculation of GMSD is shown in the following Equations.

(8) $$h_{x}=[\begin{array}{ccc} 1/3 & 0 & -1/3\\ 1/3 & 0 & -1/3\\ 1/3 & 0 & -1/3 \end{array}]\;h_{y}=\left[\begin{array}{ccc} 1/3 & 1/3 & 1/3\\ 0 & 0 & 0\\ -1/3 & -1/3 & -1/3 \end{array}\right]$$

(9) $$m_{r}\left(i\right)=\sqrt{{\left(r⊗h_{x}\right)}^{2}\left(i\right)+{\left(r⊗h_{y}\right)}^{2}\left(i\right)},$$

(10) $$m_{d}\left(i\right)=\sqrt{{\left(d⊗h_{x}\right)}^{2}\left(i\right)+{\left(d⊗h_{y}\right)}^{2}\left(i\right)}$$

(11) $$GMS\left(i\right)=\frac{2m_{r}\left(i\right)m_{d}\left(i\right)+c}{m_{r}{}^{2}\left(i\right)+m_{d}{}^{2}\left(i\right)+c},$$

where $h_{x}$, $h_{y}$ are the Prewitt operator used to calculate the image gradient. $m_{r}\left(i\right)$) and $m_{d}\left(i\right)$ are the image gradient. c is a small constant. When f+1 frame tracking fails, the GMSD measurement between the template set and the winner set is calculated to obtain the object position; the associated formula is shown in Equation (12).

(12) $$\begin{array}{c} S_{0}=\max\left(\begin{array}{c} GMS\\ 1\leq i\leq q,1\leq j\leq p \end{array}\left(C_{i},T_{j}\right)\right),C_{i}\in C,T_{j}\in T \end{array}$$

## 3. Results

We used GoogLeNet as the backbone network of SiamFC++. The number of candidate objects n=10. The number of winners q=5, filter center frequency $\;f_{0}=5$, and the band stop width =8. Our tracker is realized with PyTorch on a PC with Nvidia GTX 2080Ti, Intel(R) Core (TM) i7-7820X CPU @ 3.60GHz.

### 3.1. Dataset Description

In our experiments, OTB2015, VOT, LaSOT, and GOT-10k are adopted as our training dataset. OTB2015 Benchmark OTB2015 includes 100 videos, and VOT Benchmark VOT2018 consists of 60 videos with challenging factors such as deformation and occlusion. LaSOT is a long-term tracking dataset with 1400 video sequences, which can be divided into 70 categories. The training set of the GOT-10K dataset consists of 10,000 video sequences, which is generally a single-target tracking algorithm evaluation dataset. We followed the protocol of GOT-10k and only trained our tracker on the training subset.

### 3.2. Experiment to Verify the Effectiveness of the Arbiter

To demonstrate the effectiveness of the arbiter, the tracking invalid accuracy ratio (TIAR) on each dataset was calculated, which is the percentage of all tracking failure scenarios that were judged correctly. Moreover, in the verification experiment, the tracking valid accuracy ratio (TVAR) on each dataset was calculated, which is the percentage of all successful tracking scenarios that were judged correctly.

**Figure 9:**
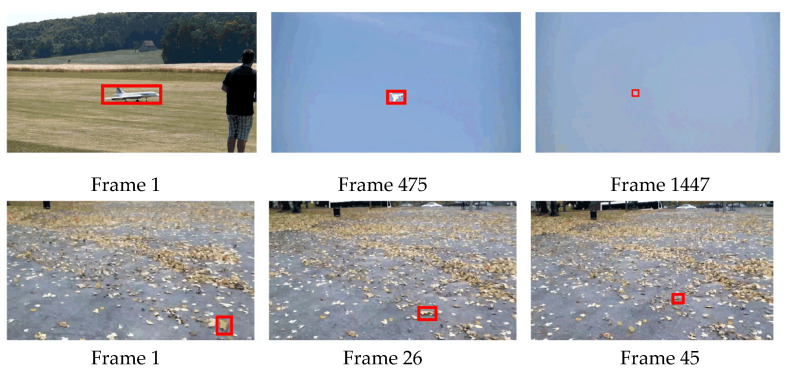
Tracking failure scenarios.

Figure 9 shows some of the scenarios where the tracking is difficult to adjudicate in different datasets, where the red borders are the target locations. The video sequence in the first row is long and the target changes too much in scale during the motion. At frame 1447, the image is so tiny that it is difficult for the tracker to recognize the target based on the depth feature alone, which makes it easy to lose the tracking and the arbiter is unable to determine whether the tracking is invalid or not. In the second row of the example, the interference in the search area is extremely similar to the target and the scale is relatively small, and slight changes in the appearance of the target during tracking can affect the tracking results.

As shown in Table 3, the arbiter can find most of the tracking failure scenarios, which is because the motion of the object is a gradual process with less abrupt changes, so the similarity score of the target is changing gradually, and when the similarity scores of the target and the interfering object are close to each other, that is, the U-shaped phenomenon occurs, the tracking failure is detected by the BRT model. The TVAR metrics in Table 2 show that there are judgment failures in scenes with successful tracking, which are due to factors such as the scale variation of targets in difficult scenes and the high similarity of interfering targets causing target frames with low confidence to shift to interferers. The TIAR difference between GOT-10k and LaSOT is large, which is due to the fact that GOT-10k is mostly short videos, and the number of image frames is about 100, so during the motion, most of the targets have small changes in appearance and scale, and the accuracy of the arbiter detection is high. The LaSOT dataset is mostly long sequences, and the number of image frames is more than 1000. Therefore, the target appearance and scale vary a lot, and more tiny targets exist. The performance of the tracker can be considered closely related to the properties of the dataset.

### 3.3. Results on Several Benchmarks

**Figure 10:**
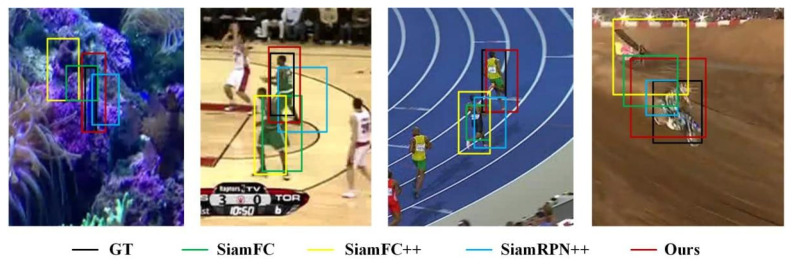
Qualitative comparison results.

We compared our proposed tracker with some of the state-of-the-art trackers on four tracking benchmarks, as shown in Table 4 and Figure 10. Our tracker obtains state-of-the-art performance. 

**Figure 11:**
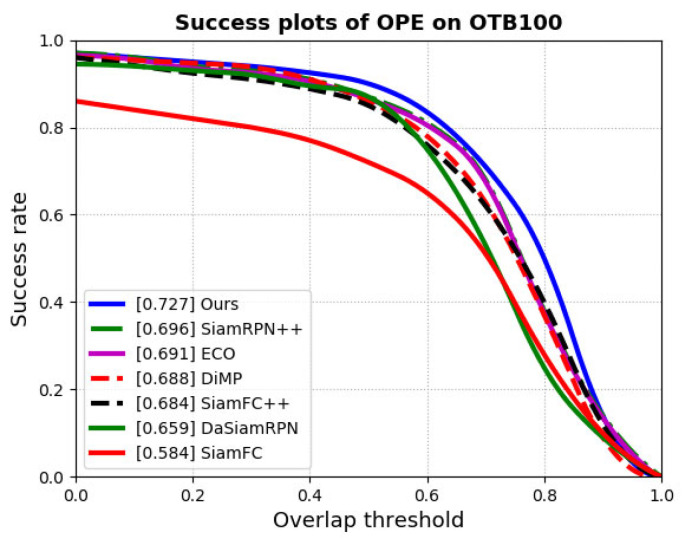
Success plots on OTB100.

**Figure 12:**
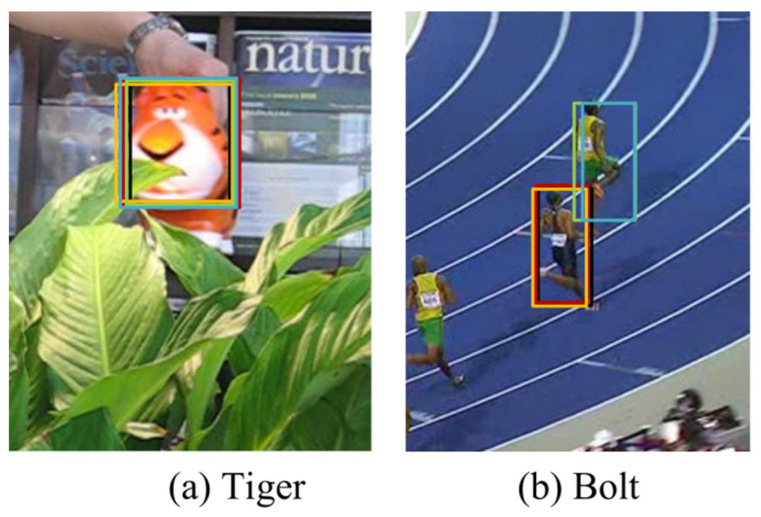
Candidate objects and correction process. In the figure, the red box represent the targets with the highest similarity scores, i.e., the tracking results, and the rest of the color boxes are the candidate targets ranked from 2 to 5.

OTB2015 Benchmark OTB2015 provides a standard evaluation benchmark for trackers. A comparison with state-of-the art trackers is shown in Figure 11 in terms of success plots of OPE. The performance of the tracker is measured by the evaluation index success score, which is defined as the percentage of successfully tracked frames whose overlap exceeds a threshold value. Our tracker obtained a success score of 0.727, which achieved state-of-the-art performance. In experiments on the VOT Benchmark, the performance of the tracker is evaluated by the accuracy. As shown in Figure 11, the bounding boxes are the candidate object with higher similarity scores and the red bounding box has the highest similarity score, which is the tracking result. In Figure 12a, when there is no distractor in the frame and the object deformation is small, the bounding boxes with high scores are concentrated on one object, and the tracking is successful at this time. When there is a distractor in the image and the tracker fails, as shown in Figure 12b, the tracking result has changed from Bolt to another athlete. At this time, some bounding box is still positioned on Bolt. By comparing the GMSD between the candidate objects and the template, the object could be found again and the tracker could be corrected. After removing the reinitialize mechanism, our tracker has the highest accuracy and obtains an accuracy score of 0.533 due to being able to relocate the object, which is a good improvement compared to SiamFC++.

LaSOT Benchmark LaSOT contains 1400 videos, which is a high-quality, large-scale dataset for long-term tracking. By relocating the lost objects, the tracker reached the top performance with a success score of 0.575, which shows that our tracker also has a good performance in difficult scenarios.

GOT-10k Benchmark GOT-10k contains a lot of small objects and they become smaller as the objects move and the viewpoint changes. It causes our tracker to fail to capture the real object when the tracker fails, which keeps the tracker from achieving a better performance.

## 4. Conclusions

In this paper, we proposed a Siamese network based on trackers with a generic arbiter-corrector module. It could resolve the tracking failure problem caused by the appearance changes of objects and distractors. The arbiter proposed an efficient architecture based on the match filter that determines whether the tracker has lost the object. The template is updated to increase the tracker’s resistance to interference. The corrector repositions the object by GMSD and dynamic template set. The generic arbiter-corrector module can be easily integrated into other trackers. The associated experiments show that the proposed arbiter-corrector mechanism is effective in improving the accuracy of the tracker. The next step of this study will introduce a self-supervised contrast learning method to construct an efficient online learning tracking corrector to further improve the tracking accuracy and real-time performance.

## Figures and Tables

**Table 1:** Challenge factors.

Challenge	Ratio
Distractor	0.3
Deformation	0.3
Scale variation	0.1
Occlusion	0.1
Other	0.2

**Table 2:** Notation table.

Notation	Meaning	Remarks
N	Length of the video frames	N.A.
$S_{j}^{i}$	j-th similarity score of the bounding Box in the i-th frame	N.A.
$R_{i}$	Bounding box set in i-th frame	$R=\{R_{1},R_{2},\;\cdot \;\cdot \;\cdot \;,\;R_{i},\;\cdot \;\cdot \;\cdot \;|\;1\;\leq \;i\;\leq \;N\}$
$D_{i}^{f}$	Candidate object set in the f-th frame	$D=\left\{D_{1}^{f},D_{2}^{f},\ldots ,D_{n}^{f}\right\}$
$D_{i}$	i-th candidate object	$D=\left\{D_{1},D_{2},\;\cdot \;\cdot \;\cdot \;,\;D_{n}\;\right\}$
$T_{p}$	p-th template	$T=\left\{T_{1},T_{2},\;\cdot \;\cdot \;\cdot \;,\;T_{p-1},T_{p}\;\right\}$
$J_{i}$	Set of tracking failure frames	$J\in \left\{J_{1},J_{2},\;\cdot \;\cdot \;\cdot \;,J_{m}\;\right\}$
n	Number of candidate objects	N.A.
q	Number of winners	N.A.
$O_{i}$	i-th center of the object	$O_{i}=\left\{x_{i},y_{i}\;\right\}$
$H_{i}$	Frequency of the i-th bin in the histogram	$H=\left\{H_{1},H_{2},\;\cdot \;\cdot \;\cdot \;,\;H_{k}\;\right\}$

**Table 3:** TIAR of the Arbiter model on several datasets.

Dataset	Number of Scenarios	TVAR	TIAR
OTB-15	100	0.86	0.68
VOT-18	60	0.81	0.59
GOT-10k	280	0.90	0.82
LaSOT	180	0.67	0.40

**Table 4:** Results on several benchmarks.

Tracker	SiamFC	ECO	SiamRPN++	ATOM	SiamFC++	Ours
OTB-15 Success	58.2	70.0	69.6	66.9	68.3	72.7
VOT-18 Accuracy [1]	0.412	0.404	0.484	0.478	0.480	0.533
LaSOT Success	33.6	32.4	49.6	51.5	54.5	57.5
GOT-10k AO	34.8	31.6	51.8	55.6	59.5	61.2

## Data Availability

Not applicable.
